# A Measurement Method of Power Transferred to an Electric Vehicle Using Wireless Charging

**DOI:** 10.3390/s23249636

**Published:** 2023-12-05

**Authors:** Žilvinas Nakutis, Robertas Lukočius, Viktoras Girdenis, Kaspars Kroičs

**Affiliations:** 1 Faculty of Electrical and Electronics Engineering, Department of Electronics Engineering, Kaunas University of Technology, Studentų St. 50, 51368 Kaunas, Lithuania; 2 Faculty of Electrical and Electronics Engineering, Department of Electrical Power Systems, Kaunas University of Technology, Studentų St. 48, 51367 Kaunas, Lithuania; robertas.lukocius@ktu.lt; 3 Institute of Industrial Electronics and Electrical Engineering, Riga Technical University, 12/1 Azenes St., 1048 Riga, Latvia; kaspars.kroics@rtu.lv

**Keywords:** power measurement, electric vehicles, wireless charging, measurement error

## Abstract

The increasing number of zero-emission vehicles on the roads demands novel vehicle charging solutions that ensure convenience, safety, increased charging infrastructure availability, and aesthetics. Wireless charging technology is seen as the one that could assure these desirable properties and could be applied not just in conventional implementations but also in off-grid solutions together with roadway energy harvesting systems. Both approaches require proper transfer of energy metering methods. In this paper, a method for measuring the power transferred to the load in a wireless charging system is presented, and its systematic error is assessed in the relevant range of influencing factors. The novelty of the method is that it does not require any metrologically certified measurement instrumentation on the receiver side of the wireless charging system. The error analysis is performed using a numerical simulation. Considered error-influencing factors included secondary side electrical load, coils’ coupling coefficient and quality factor, current and voltage quantization resolution, and compensation topology type (serial-serial (SS) and serial-parallel (SP)). It was determined that the systematic error of the power assessment does not exceed 0.7% for SS and 1.1% for SP topologies when the coupling coefficient is in the range of 0.05 to 0.4 and the quality factor of the resonant system is in the range of 100 to 800.

## 1. Introduction

Wireless charging of electrical vehicles (EVs) in stationary or dynamic conditions is gaining more popularity [1,2,3,4]. The EV market has been growing rapidly in recent years. This growth is expected to boost the size of the global wireless charging market for EV charging applications, too. Wireless charging market size is projected to grow from 80 million USD in 2023 to 1279 million USD by 2030, exhibiting a compound annual growth rate of 48.4%, according to [5]. Since wireless charging is hands-free, it significantly increases the convenience of the charging process and user satisfaction. The market will be additionally boosted by its synergy with Vehicle-to-Grid (V2G) technology and by the growth of autonomous and semi-autonomous EVs. Unburdening connection/disconnection to a power grid makes V2G technology much more attractive and accessible. Wireless EV charging and autonomous, semi-autonomous EV features such as self-parking are seen as complementary factors, which could further enhance drivers’ satisfaction and promote the technology [5]. Static and dynamic wireless charging systems could be integrated into a city’s infrastructure without any influence on its aesthetics and applied to both public and personal vehicles. Many efforts have already been put in and are still in progress to analyze and optimize configurations of charging coils, power electronics, and compensation topologies for EV charging applications [6,7]. Compensation circuits are used in magnetic resonance coupling-based wireless power transfer systems, contrary to those based on electromagnetic inductance. Compensation circuits are dedicated to maintaining resonance conditions that enable higher power transfer capability and efficiency in the system. The efficiency improvement is particularly noticeable if the energy is transferred through large air gaps, which are typical in EVs charging applications. Different structures of compensation circuits (compensation topologies) are used to determine the different properties of wireless power transfer systems [8]. The released standards (e.g., SAE J2954, IEC TS 61980-3:2019) facilitate faster adoption of WPT technology in e-transport infrastructure [4,9]. Stationary and dynamic wireless EV charging brings a lot of benefits [10,11,12], is a promising technology for conventional grid-connected solutions, and is an essential part of Future Sustainable Roads for Electric Mobility, combining energy harvesting and other modern technologies [13,14]. As we approach the end of the period of subsidizing electrical energy for EV charging, the issue of fair billing in both contact and wireless charging systems is getting more attention. Initially, AC meters at the electricity grid connection point were used to measure the amount of energy for consumer (EV owner) billing. In fair trading, a consumer should not be taxed for the energy due to technical losses at the charging station (CS). The energy dissipated into the ambience due to the misalignment of EVs with respect to charging installation should not be included in the bill, either. On the other hand, conversion losses appearing on-board of EVs or due to the EVs presence close to the charging zone can be fairly attributed to the bill of energy purchase. In traditional or emerging advanced billing schemes [15], measurement methods of the energy delivered to the consumer load (EV) become of paramount importance. It is not reasonable to argue that consumers will need to pay for all losses either directly or as a result of increased tariffs by the energy provider. Indeed, the payment for loss should follow the principle that the consumer acquiring a larger quantity of energy has to pay more for the losses of the technology used in place. For that reason, measurement techniques disaggregating provider-related losses and consumer-delivered energy must be implemented. It is obvious that electrical losses may be dependent on the ambient conditions (temperature, humidity), status and features (cable losses, switching) of CS, current and voltage during charging, etc. Therefore, the CSs loss characterization cannot be conducted in advance and then subtracted from the total energy measured at the input of the station.

Some analysis was already presented for the US market [16], demonstrating that even a 1% error in measuring transferred energy in wireless charging systems would amount to 1 billion USD in unfair billing by 2030. Therefore, research on methods and techniques to accurately measure only the energy transferred to the receiver has attracted the focus of research teams recently.

Metrological certification of measurement equipment used for billed energy measurement is mandatory and dependent on every country’s national legal trade regulation system. If part of the metering equipment is located in an EV, the process of energy meter verification may become difficult (most probably during EV technical inspection), and the cost of metrological supervision is very high due to the large fleet of EVs. Therefore, it is highly preferable that all the energy measurement instrumentation is located in the CS, similarly to the traditional implementation of petrol stations, which are equipped with fuel flow meters. This means that power delivered and consumed in the EV should be sensed using only current and voltage meters assembled on the primary (transmitter) side.

The EURAMET MICEV project (https://micev.eu, accessed on 30 November 2023) was dedicated to the metrology of WPT systems for EV charging. In particular, a measurement system for the on-site characterization of wireless charging stations was developed that is capable of verifying its efficiency by measuring the power supplied and propagated from the electricity grid to the AC/DC converter, the DC/AC converter, the transmitter coil, the receiver coil, the AC/DC converter, and the DC/DC converter feeding the battery of an EV [17]. Nevertheless, the project did not target techniques that could be used to measure the power delivered to the EV side without measuring current and voltage on the EV board.

The aim of this research is to suggest a new active transferred energy measurement method utilizing only measurements (current and voltage) conducted at the transmitter side. Also, it was aimed at establishing current and voltage sensors in the high-frequency circuits of transmitters, seeking to exclude losses in the power electronics circuits of the CS (namely losses of power electronics converters) from the total transferred energy. Calibration or verification of the provider’s measurement equipment installed in the stationary CS is preferable compared to metrological verification of the energy meter installed in the EV. Estimating an achievable energy measurement error is the second objective that enables us to specify the performance of the proposed technique.

The rest of this paper is organized as follows: A review of the related works is available in Section 2. The applied research methodology is shown in Section 3. The proposed method is presented in Section 4. Details of the applied analysis for determination of the method’s accuracy are explained in Section 5, and the results of the analysis are presented in Section 6. The aim of the method, its novelty, and its benefits for consumers are presented in Section 7. The results and findings of the work are generalized, and directions for future work are presented in Conclusions (Section 8).

## 2. Review of Related Works

Different transmitted energy measurement points in the WPC systems are discussed in [18]. The so-called ground-side and vehicle-side billing modes are distinguished. Concerning the ground-side energy metering of the AC grid and transmitting coil points, measurement modes are possible. The fairest location to measure transferred energy is ground-side transmitting coil measurement [18], because charging pile losses are not attributed to the bill of the EV owner. Challenges related to the ground-side transmitting coil measurement mode are due to relatively few studies of power measurement at high frequencies and the absence of standard equipment. The method that we propose is dedicated to contributing to the research of ground-side transmitting coil measurement techniques. Among the techniques published so far, there is only the series of works by Chu et al. [16,19] that explore the method of measuring transmitted power at high frequency by installing two additional field sensing coils between the ground coil and receiver coil. Our technique, as described later, does not demand any additional coils and, in this sense, is less demanding for installation cost. This advantage should become even more critical in dynamic charging on the roads with built-in coils because assembling separate sensing coils would impose technical challenges and a cost burden. The transferred energy metering in dynamic WPC is also in the very initial stage of the research field. The study [20] considered fundamental frequency AC metering for energy delivered to moving EVs. Any research on dynamic wireless power transfer metering at high frequencies has not yet been investigated to the best of our knowledge.

In [16], the authors present a non-contact method of measuring the transfer power over the air gap from the transmitter to the receiver using two Faraday coils assembled in the space between the WPT Tx and Rx coils. Faraday transfer-power measurement coils are open-circuit and do not consume power from the transmitter. Voltage meters (commercial off-the-shelf analog-to-digital converters) are connected to the output of each sense coil for the acquisition of the induced voltage. The achieved transfer-power measurement accuracy was stated not to exceed 0.1% in the range of powers from 60 W to 1 kW. Sensing coil installation accuracy can be eliminated by the calibration process, which is not yet described in detail. The authors plan to advance their research in terms of estimating the influence of multidimensional variations, including various types of charging system geometries [16]. Weaknesses of the technique include the necessity to install two additional sense coils and the absence of established calibration and verification procedures for the measurement setup.

A concept of reflected impedance from the receiver circuit to the transmitter circuit is suggested to estimate the distance between WPT system Tx and Rx coils in [21]. The reference does not target transferred power measurement, but the technique may be transformed by measuring reflected impedance in resonance conditions and then estimating the power consumed in the real part of the reflected impedance. It was assumed that the power dissipated in the real part of the reflected impedance is a rather accurate approximation of the power delivered to the load of the receiver output. This idea was first suggested in [22].

In [23], the authors target determining the secondary coil load by sensing the DC current and voltage of the inverter driving the WPT system transmitter. They show that the tangent value of the reflected impedance angle and the ohmic load is linearly monotonic. Though measurements are conducted only on the primary side, where metrological certification of meters is acceptable, the transferred power to the determined load is not investigated in this paper.

**Figure 1:**
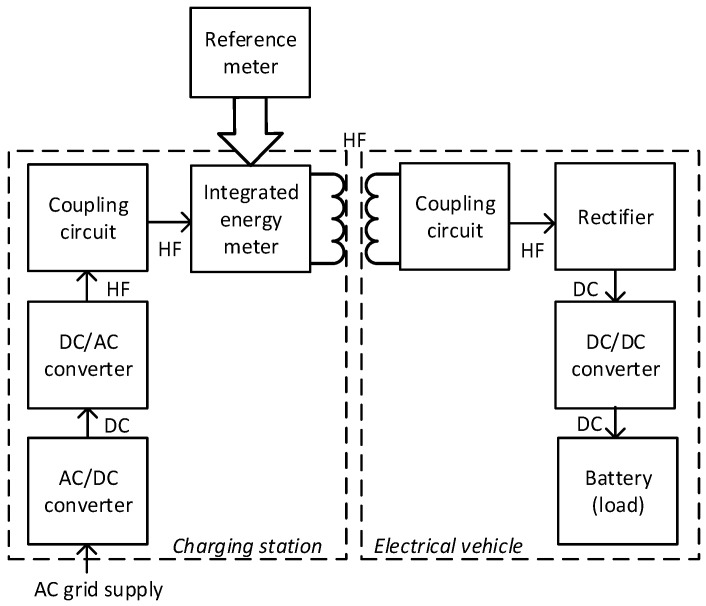
Expected energy meter integration in CS and certified meter-free EV. The metrological reference meter shown is only required during metrological verification. DC—direct current; HF—high frequency circuits.

Aiming to measure the energy delivered to an EV and bill the owner accordingly, the traditional approach is based on the measurement of AC energy supplied to the CS for the period of charging. However, the WPT CS transmitter side energy losses are beyond the responsibility of the EV owner and preferably should be excluded from the total supplied energy when calculating the price of the charging transaction. Measurement of energy losses or efficiency of WPT systems was studied and reported extensively in [24,25,26,27,28,29]. All these techniques focus on estimating losses using instrumentation for current and voltage measurement at both transmitter and receiver circuits. They may be suitable for metrological verification of the integrated energy meter of a CS. Inevitably, during the metrological verification of the CS, the reference meter will be connected at some point in the energy flow path in order to compare its readings with the readings of the CSs energy meter (Figure 1). However, the integrated meter alone is expected to estimate the transmitted power, i.e., indirectly or directly; it has to account for the losses emerging in the CS. Being connected as close as possible to the transmitting coil (behind the power electronics of all converters), as shown in Figure 1, it will automatically measure transmitted power, which does not include energy losses due to the transmitter’s power electronics. However, energy losses emerging in the transmitting resonant circuit must be estimated and subtracted from the energy measured in the high-frequency resonant circuit of the WPT system transmitter.

To conclude the overview of solutions suggested in publications, it may be confirmed that any well-established method suitable for measuring power (energy) delivered to the EV, excluding losses of CS (provider infrastructure) and not demanding metrologically certified meters on-board the EV, is absent to the best of our knowledge. It is believed that the implementation of such a method could ensure both a fair billing of consumers and the minimization of metrological supervision costs.

## 3. Research Methodology

In the initial stage of a new measurement technique concept development, a simulation-based approach is preferable, seeking to avoid any influence from interfering factors that would have been challenging to control and quantify in a physical setup. Features of hardware dedicated to primary coil driving (power electronics), imprecise knowledge of the physical parameters of coils and their positioning, and measuring circuit quality and performance (noise, drifts, errors, etc.) inevitably contribute to the quality of experimentally acquired data. Therefore, aiming to preliminary explore the proposed technique in a purified environment free from any unexpected influence from external factors, it has been followed by a simulation using Simulink, which is de facto standard in different domains. Implementation of a model of the WPC system enabled the setting of parameters of interest precisely according to the chosen plan, in contrast to coping with challenges using physical prototyping. Several compensation circuit topologies are achievable to investigate with much less cost compared to the methodology, including the manufacturing of the hardware.

Wireless transfer coils’ coupling with external components and circuits is possible using several known topologies. So-called serial-serial (SS) and serial-parallel (SP) compensation topologies are among the most simple and applicable. Magnetic resonant coupling (MRC) became a prevailing technology for wireless power charging of EVs because of its better efficiency at larger distances between coils compared to inductive power charging [6,9,30,31]. Therefore, further focus is set on MRC WPT.

The suggested measurement method’s power measurement error analysis is performed using simulations of WPT systems utilizing SS or SP compensation topologies. Since it is expected that the energy (or power) measurement error depends on various influencing factors, it will be sought to determine the largest error in the most probable (from a practical application point of view) range of the identified influencing factors. Though the error might be less in other parts of the investigated range of influencing factors, the fair and uncomplicated way to characterize the method’s characterization is by specifying the worst-case (maximum) error.

The typical range of the coupling coefficient for EV wireless charging applications is 0.2 ≤ *k* ≤ 0.3 [32]. However, the performance of the method was investigated in a slightly wider range of 0.05 ≤ *k* ≤ 0.4 to bring a broader perspective about its robustness, even in some possible non-typical cases such as charging of EVs with non-standard, small ground clearance (extended upper *k* range case) or considering the effect of coils’ misalignment (extended lower *k* range case). Anyway, discussions about the performance of the method in typical ranges of the coupling coefficient associated with EV wireless charging will be provided as well. To maximize efficiency, which is crucial in such high-power applications as EV charging, the coils must be designed to have a high quality factor. The quality factors of the coils for WPT applications could reach a few hundred [6,33,34,35], but only the coils with quality factors no less than 100 are considered suitable for WPC applications [36]. Therefore, analysis was performed in a wide quality factor range, starting from *Q* = 100 up to *Q* = 800.

## 4. A Method for Transferred Power Measurement

### 4.1. Power Transfer Modeling Using Reflected Impedance

**Figure 2:**
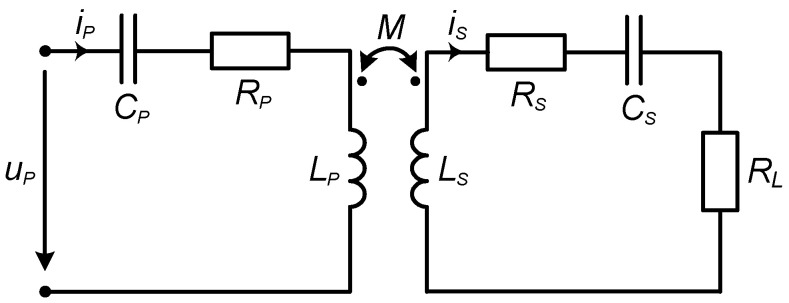
Circuit of the WPT system. SS compensation topology.

We consider the MRC transfer system implemented using mutually coupled coils (Figure 2). The schematics of the system are shown in Figure 2, where the subscript p denotes the circuit voltage and current on the primary (transmitter) side, and the subscript s stands for the secondary (receiver) side voltage and current. The concept of reflected impedance is widely used in the circuit analysis of mutually coupled coil systems [37]. Reflected impedance, in inductive coupling systems, is the equivalent impedance of the secondary side of the coupling, including the load impedance, as seen from the primary side. The influence of secondary side components on the primary circuit is modeled by the reflected impedance connected in series to the primary side coil, despite the compensation topology in use.

**Figure 3:**
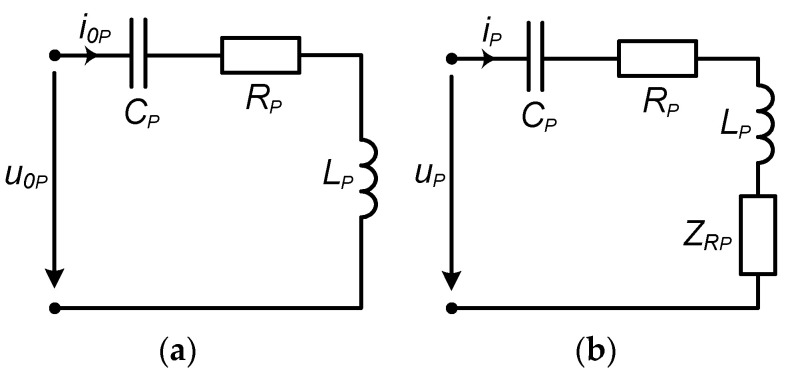
The equivalent circuit of the WPT system primary circuit is in (**a**) no-load mode and (**b**) loaded mode.

In EV charging systems implementing WPT, it is typical that the secondary side (assembled on the mobile EV) location is not fixed. In cases where the receiver side has not yet approached the charging point (primary side), the reflected impedance is absent (Figure 3a), and such an operation mode is called no-load. When the EV with the receiving coil drives close to the charging spot, the reflected impedance becomes noticeable and dependent on the distance and position of the receiver coil with respect to the transmitter coil. This mode is called the loaded mode (Figure 3b).

The active power transferred to the secondary side is modeled by the real part of the reflected impedance and current in the primary coil, according to Figure 3b.

(1) $$P={Re(Z}_{Rp})\cdot I_{P}^{2},$$

where $I_{P}$ is the current root-mean-square (RMS) value in the primary circuit, and Re() denotes the real part of the complex argument.

Since the primary side excitation voltage may be non-sinusoidal (typically rectangular) or the secondary side load *R_L_* may be non-linear (for instance, a rectifier circuit input), the total active power is transferred by different harmonics

(2) $$P_{\Sigma }=\sum_{h=1}^{N}{Re(Z_{Rp(h)})I}_{P(h)}^{2},$$

where $Z_{Rp(h)}$ is the reflected impedance value at the frequency of the *h*th harmonic and the $I_{P(h)}$ is the current RMS value of the *h*th harmonic.

The value of reflected impedance at different harmonics’ frequencies may be different due to skin and proximity effects [38].

### 4.2. Transferred Power MeasurementProcedure

The suggested method of transferring active power measurement involves two steps. First, the primary coil current $i_{0p}$ and voltage $u_{0p}$ are measured in no-load mode (EV is far away and the secondary coil and load are absent).

Since in general the excitation voltage may be non-sinusoidal or due to a nonlinear load, a group of voltage and/or current harmonics contribute to power transfer. The current and voltage waveforms are sampled, and FFT is applied to estimate complex harmonics $I_{0p(h)}$ and $U_{0p(h)}$ of interest. The no-load impedance of the *h*th harmonic is then calculated

(3) $$Z_{0p(h)}=\frac{U_{0p(h)}}{I_{0p(h)}},\;h=\bar{1,N},$$

where $\bar{1,N}$ denotes the considered range of the harmonics from 1st to *N*th.

In practical implementation, the driving voltage must be reduced in order to prevent overcurrent in the primary circuit operating in resonant conditions [3]. Seeking a higher accuracy of WPT system primary side impedance estimation, it should be measured at a temperature that is reached by driving with nominal current, similarly to that reported in [39].

When an EV with a secondary coil approaches the charging point, the primary coil current $i_{Lp}$ and voltage $u_{Lp}$ waveforms are measured in order to estimate the primary side impedances corresponding to the load mode conditions according to the expression

(4) $$Z_{Lp(h)}=\frac{U_{Lp(h)}}{I_{Lp(h)}},\;h=\bar{1,N},$$

where $I_{Lp(h)}$ and $U_{Lp(h)}$ are complex values of current and voltage harmonics estimated using FFT from sampled current and voltage waveforms.

As it is expected, skin and proximity effects become noticeable for the excitation frequency (85 kHz) and its harmonics, $Z_{0p(h)}$ and $Z_{Lp(h)}$ must be estimated for each harmonic individually.

The reflected impedance for the *h*th harmonic is defined by the expression [40].

(5) $$Z_{Rp(h)}=Z_{Lp(h)}-Z_{0p\left(h\right)}.$$

The active power consumption of the reflected impedance transferred by the *h*th harmonic can be expressed

(6) $$P_{2(h)}^{\prime }={\left|I_{Lp(h)}\right|}^{2}\cdot Re\left(Z_{Rp(h)}\right),h=\bar{1,N}.$$

The corresponding total power consumption is

(7) $$P_{2}^{\prime }=\sum_{h=1}^{N}P_{2(h)}^{\prime }.$$

Referring to Figure 3b,

(8) $$Re\left(Z_{Rp(h)}\right)=Re\left(Z_{Lp(h)}\right)-Re\left(Z_{0p\left(h\right)}\right).$$

The real part of no-load impedance can also be described

(9) $$Re\left(Z_{0p\left(h\right)}\right)=\left|Z_{0p\left(h\right)}\right|\cdot cos\left(arg\left(Z_{0p\left(h\right)}\right)\right),$$

(10) $$Re\left(Z_{Lp\left(h\right)}\right)=\left|Z_{Lp\left(h\right)}\right|\cdot cos\left(arg\left(Z_{Lp\left(h\right)}\right)\right),$$

where arg() denotes the angle of a complex argument.

Expressions (3)–(10) are valid for both SS and SP WPT system topologies. In SS topology, the transmitter circuit reactance is close to zero because it remains tuned to the resonance in both load and no-load conditions. On the other hand, in the case of SP topology, the resonance conditions are achieved only in load mode [30]. When the EV carrying the load departs from the transmitter coil, the primary circuit exits the resonance state.

Despite the topology used, $Re\left(Z_{0p(h)}\right)$ can be determined using (9). However, it was found later that the accuracy of its assessment is less influenced by errors of current and voltage magnitudes and phases in the case of resonance conditions. Therefore, it is favorable to ensure resonance conditions in the transmitter circuit during the estimation of $Re\left(Z_{0p(h)}\right)$. In the case of the SP topology, this can be achieved either by changing the excitation frequency or by switching on an additional capacitor. However, inserting an additional component will change the losses in the transmitter circuit. Temporarily tuning the frequency is expected to cause lower errors on the transferred active power. Therefore, the last approach was chosen. Though it may be in conflict with the standard frequency allocated by standards [30], The power of the excitation frequency for the moment of determining $Re\left(Z_{0p(h)}\right)$ may be reduced to mitigate EMC requirements.

To implement the proposed solution, the primary side current $I_{0p}$ and voltage $U_{0p}$ must be measured. Primary side current is usually measured and controlled by the WPT controller in order to control power, avoid overload, and limit reactive power [41]. Since a resonant network operates as a filter, this current is nearly sinusoidal, and the noise level is not high. The voltage at the inverter output is usually not measured. Frequency control, phase shift, pulse density, and DC voltage control are the most often used control methods [42]. Depending on the control method, the shape of the voltage waveform might be different. Since the control signals are generated with the same microcontroller, in some cases, the voltage waveform can be predicted without measurement. For measurement, a simple resistive or capacitive voltage divider can be used, and additional analog and digital filtering can be applied to avoid high-frequency noise. The voltage signal could be noisy, with fast rising and falling edges, so for more precise measurement, a harmonic filter similar to that in [43] can be developed and added to the circuit.

## 5. The Proposed Measurement Method: Accuracy Analysis

**Figure 4:**
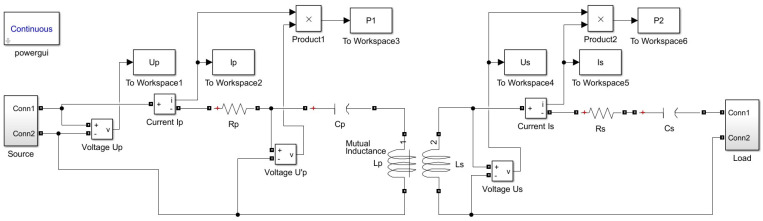
WPT system Simulink model.

**Figure 5:**
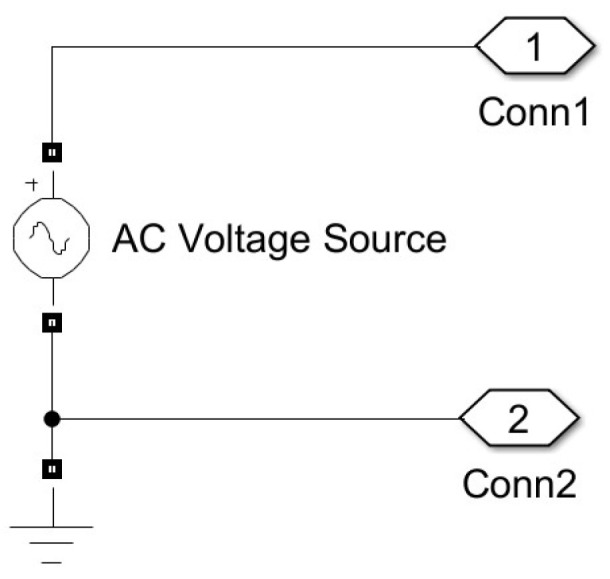
Simulink model of the sinusoidal source.

**Figure 6:**
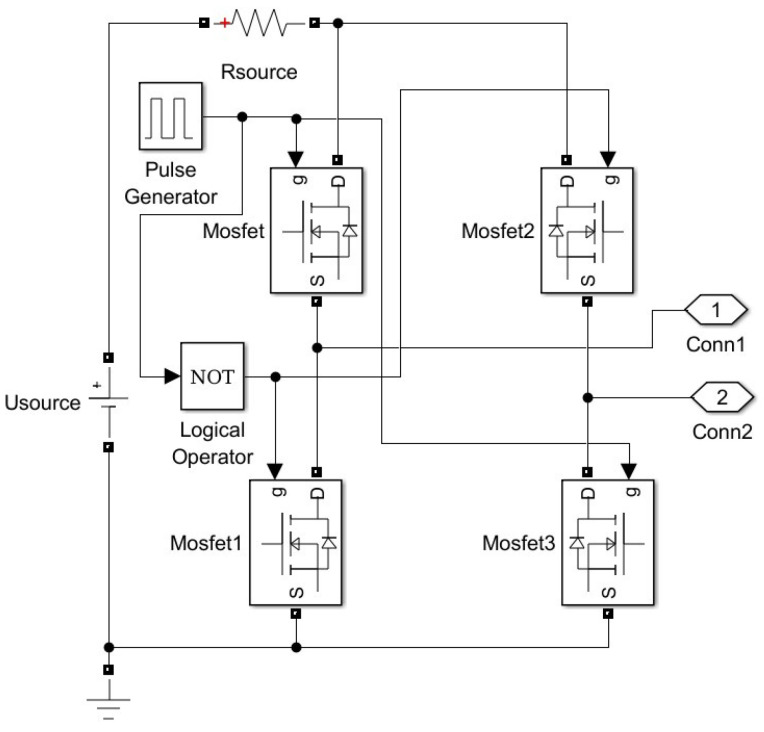
Simulink model of the square wave source.

**Figure 7:**
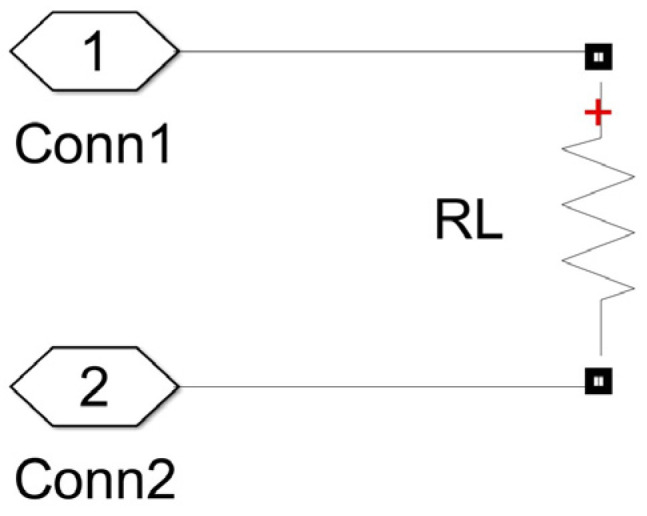
Simulink model of the linear load.

**Figure 8:**
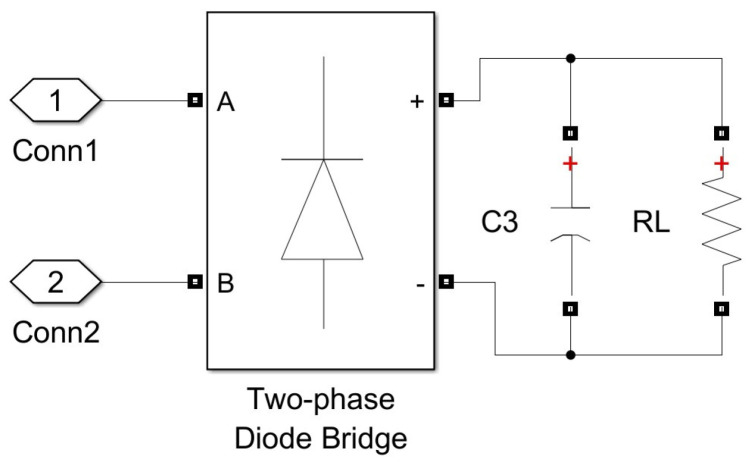
Simulink model of the non-linear load.

Analysis of the method’s accuracy was conducted for both SS and SP compensation topologies using Matlab/Simulink modeling. The model of the WPT system implemented in Simulink is presented in Figure 4 and includes typical wireless coil driving and loading electronics [4,6,44]. The two-phase mutual inductance component is used from the Simscape library, and it is characterized by parameters namely primary and secondary coil inductance $L_{p}$ and $L_{s}$ respectively, and coupling coefficient *k* [45]. As the resistances of the WPT system transmitting and receiving coils were modeled by the external resistances $R_{p}$ and $R_{s}$ (Figure 4), the internal resistances of the mutual inductance component were set to zero. The values of the resistances $R_{p}$ and $R_{s}$ in particular implementation could vary upon system design and operation conditions. The characteristic values of the resistances for the WPT system were chosen from [46]. The number of harmonics N considered in this research, as well as the sampling frequency, $f_{s}$ was determined by applying the sensitivity analysis. The number of harmonics was determined by changing its value and analyzing its impact on the method’s error. The analysis showed that the application of a higher number N>3 of harmonics does not reasonably increase the accuracy of the method. Sampling frequency, $f_{s}$ was determined in the same way. It was picked as small as possible but high enough to ensure acceptable values for the method’s error. The source module was implemented according to the schematics shown in Figure 5 (sinusoidal source) or Figure 6 (square wave source). The square wave source in the model is implemented by the direct voltage source, representing rectified voltage, and the full-bridge metal oxide semiconductor field effect transistors (MOSFET) inverter [47], driven by signals from the pulse generator [24,28,31,32,36,40,48]. The load module was implemented according to the schematics shown in Figure 7 (linear load) or Figure 8 (non-linear load). The non-linear load in the model is represented by a two-phase diode bridge [49], a smoothing capacitor, and DC side load resistance [24,31,50,51]. Voltage and current meters in Figure 4, Figure 5, Figure 6, Figure 7 and Figure 8 were used to measure corresponding voltage and current signals.

Error of transferred power assessment using the presented method was investigated for a wide range of coupling coefficient $k=M/\sqrt{L_{p}L_{s}}$ values, for a wide range of load resistance values $R_{L}$, in a wide range of the coils’ quality factor $Q=Q_{p}=Q_{s}=\frac{\omega L_{p}}{R_{p}}=\frac{\omega L_{s}}{R_{s}}$ values, where M is mutual inductance, $L_{p}$ and $L_{s}$ are inductances of the primary and secondary coils, $R_{p}$ and $R_{s}$ are resistances of the primary and secondary coils correspondingly, ω is angular frequency. The values of the resistances and the angular frequency were set constant. Inductances $L_{p}$ and $L_{s}$ for particular quality factor $Q=Q_{p}=Q_{s}$ value were calculated applying the expressions provided in Table 1. The chosen ranges of the coupling coefficient and quality factor are presented and explained further in the text (in this Research section). Capacitances of compensation circuity capacitors $C_{p}$ and $C_{s}$ were calculated by the formulas presented in Table 1. Operation frequency f=85 kHz applied in the analysis is common for standardized charging power levels used for EV charging applications [3] and is defined by the standard SAE J2954 [31]. The values of the parameters used in the simulations and/or expressions for their calculations are summarized in Table 1.

The transferred relative active power measurement error was estimated according to the expression

(11) $$\delta P=\frac{P_{2}^{\prime }-P_{2}}{P_{2}}\cdot 100\%,$$

where $P_{2}^{\prime }$ is transferred power calculated applying the method and $P_{2}$ is power consumed at the secondary winding obtained using digital integration of the instantaneous power according to expression

(12) $$P_{2}=\frac{1}{K}\sum_{i=1}^{F}p_{2i}=\frac{1}{K}\sum_{i=1}^{F}u_{si}i_{2i},$$

where *K* is the number of complete periods considered (*K* = 15 applied), and *F* is the number of samples per the considered number of full periods $F=K\cdot f_{s}/f$. The value of the parameter was determined by applying its sensitivity to the method’s error analysis.

Analysis of the method’s accuracy was investigated for the following driving source and load combinations: (1) the driving source is sinusoidal and the load is linear (Figure 7); (2) the output of the driving source is square wave and the load is non-linear (Figure 8). The first combination analysis was aimed at verifying the power measurement errors occurring due to the simulation precision. The second combination analysis is closer to the real implementation of WPT setups in EV charging spots. The analysis of the error does not involve errors in current and voltage sensors, which should be included in the final uncertainty budget.

The influence of quantization of the current and voltage waveforms is explored as well.

## 6. The Results

### 6.1. SS Compensation Topology

**Figure 9:**
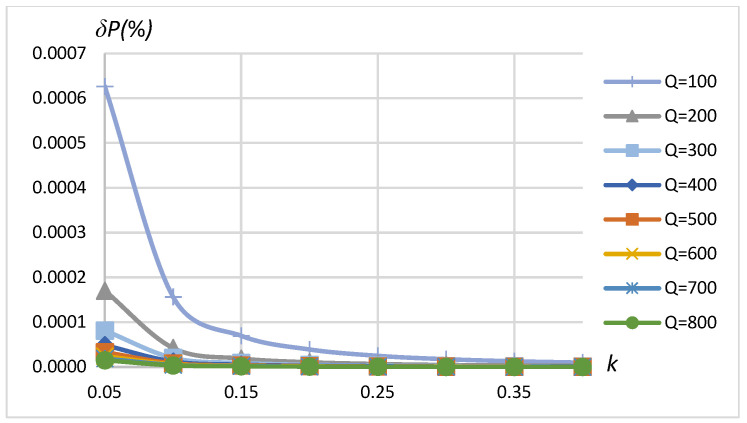
*δP* vs. coupling coefficient *k* and vs. quality factor *Q*. SS compensation topology, sinusoidal source, linear load ($R_{L}=75\;\Omega$).

The obtained dependencies of the active power measurement error δP as a function of coupling coefficient k for a selected set of Q values when the source is sinusoidal and the load is linear are presented in Figure 9. The results show that the error in the ranges of coupling coefficient 0.05≤k≤0.4 and quality factor 100≤Q≤800 of the method is less than $7\times 10^{-4}\%$. In the typical range of k=0.2÷0.3 for EV wireless charging applications [32], it is less than $4\times 10^{-5}\%.$

This level of power estimation error is sufficient for practical applications and is much below the requirements of standards. Therefore, it was assumed that the methodology involving the presented schematics of the WPT system and the precision of modeling using Simulink are suitable for the analysis of the error of active transferred power in a wireless charging arrangement.

**Figure 10:**
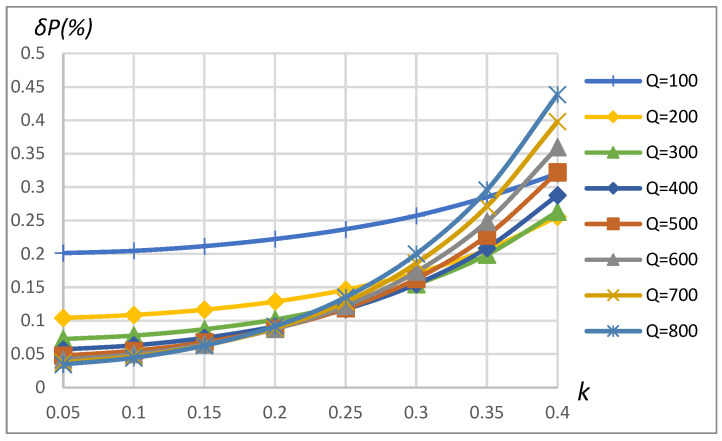
*δP* vs. coupling coefficient *k* and vs. quality factor *Q*. SS compensation topology, rectangular source, non-linear load.

**Figure 11:**
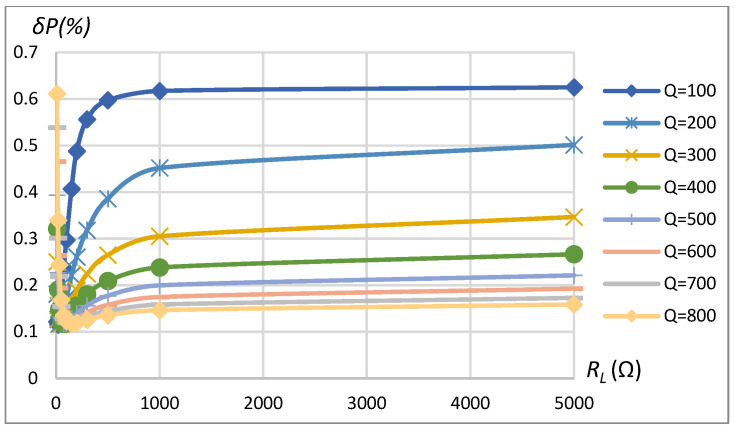
*δP* vs. load resistance *R_L_* and vs. quality factor *Q*. SS compensation topology, rectangular source, non-linear load.

Figure 10 and Figure 11 correspondingly present power measurement error dependence on *k* and *Q* in the case of driving the primary coil with the rectangular shape waveform and modeling the non-linear load according to the schematics shown in Figure 8. It can be seen that the error may reach up to 0.45% (Figure 10) and 0.65% (Figure 11), depending on the coupling coefficient, coil’s quality factor, and load values. The increase in the method’s error compared to the sinusoidal driving source and the linear load is caused by the influence of current and voltage harmonics on errors of no-load and reflected impedance real part assessment according to (9) and (10) correspondingly using magnitude and phase estimates obtained using FFT (expr. (3) and (4)).

The results show that the error of the method is less than 0.45% in the range of k=0.05÷0.4 and is less than 0.25% in the typical coupling coefficient range k=0.2÷0.3 for EV wireless charging applications. It is visible from the error’s higher sensitivity to the coupling coefficient for higher quality factor values.

### 6.2. SP Compencsation Topology

**Figure 12:**
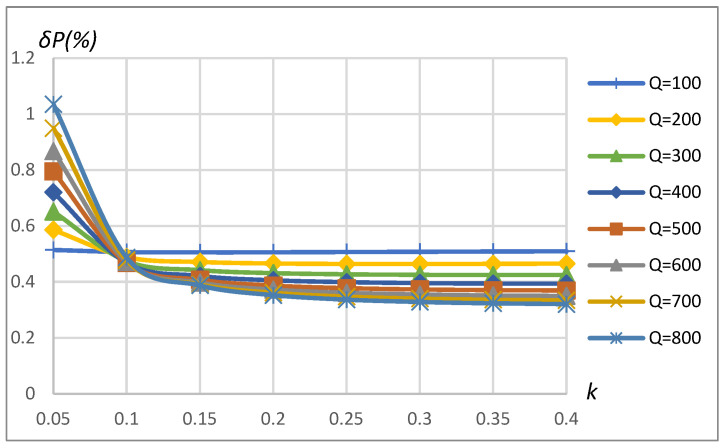
*δP* vs. coupling coefficient *k* and vs. quality factor *Q*. SP compensation topology, rectangular source, non-linear load.

**Figure 13:**
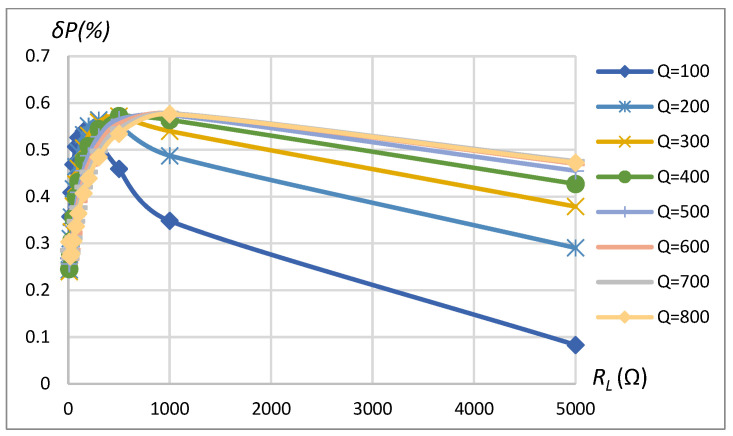
*δP* vs. load resistance *R_L_* and vs. quality factor *Q*. SP compensation topology, rectangular source, non-linear load.

A similar analysis was repeated for the SP compensation topology of the WPT system using the parameters specified in Table 1. The summarizing table of the method’s maximum power assessment error dependence on the range of influencing factors is given in Table 2. Sinusoidal driving of a linear load exhibited a power assessment error less than 0.012% in the range of considered *k*, *Q*, and $R_{L}=75\;\Omega$. If the load is increased to $R_{L}=10\;\Omega ,$ the maximum error of power assessment is not more than 0.03%. However, the rectangular-shaped driving and non-linear type of load resulted in errors reaching up to 1.1% in the case of low coupling coefficients *k* = 0.05 (see Figure 12) and up to 0.6% in the case of higher loads (lower $R_{L}$) as can be seen from Figure 13. The analysis reveals that power assessment errors are slightly higher in the SP topology compared to the SS topology.

### 6.3. Quantization Resolution Influence on Power Assessment Error

Since practically in all modern power measurement implementations, sampling and quantization of current and voltage signals are performed using ADC prior to processing in the digital domain, the analysis of the influence of the resolution of quantization on the assessment error of transferred power is relevant. The analysis was performed for the cases of rectangular driving and non-linear loads. Voltage and current signals were quantized by an ideal ADC with *n*-bit resolution.

(13) $$i_{1Q}=round\left(\frac{G_{i}\cdot \left(i_{1}+i_{range}/2\right)}{{∆}_{i}}\right)\cdot \frac{{∆}_{i}}{G_{i}}-i_{range}/2,$$

(14) $$u_{1Q}=round\left(\frac{G_{u}\cdot \left(u_{1}+u_{range}/2\right)}{{∆}_{u}}\right)\cdot \frac{{∆}_{u}}{G_{u}}-u_{range}/2,$$

where current and voltage amplifiers’ gains are

(15) $$G_{i}=i_{ref}/\left(i_{range}\right),$$

(16) $$G_{u}=u_{ref}/\left(u_{range}\right),$$

where $i_{ref}$ and $u_{ref}$ are ADC reference levels in the current and voltage sensing channels, and quantization steps are correspondingly

(17) $${∆}_{i}=i_{ref}/(2^{n}-1),$$

(18) $${∆}_{u}{=u}_{ref}/(2^{n}-1),$$

where $i_{range}$ and $u_{range}$ are peak-to-peak range of respectively current and voltage in the particular amplification interval:

(19) $$i_{range}=k_{range}\cdot 2\cdot max\left(i_{1}\right),$$

(20) $$u_{range}=k_{range}\cdot 2\cdot max\left(u_{1}\right),$$

where the range coefficient is $k_{range}=4/3$.

**Figure 14:**
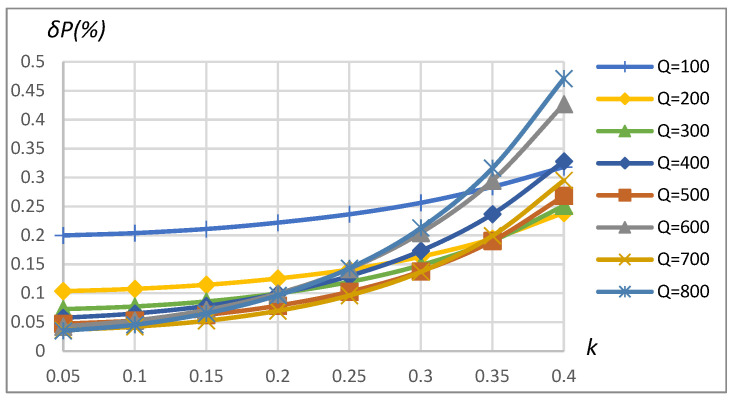
*δP* vs. coupling coefficient *k* and vs. quality factor *Q*. SS compensation topology, rectangular source, non-linear load, 16-bit ADC.

**Figure 15:**
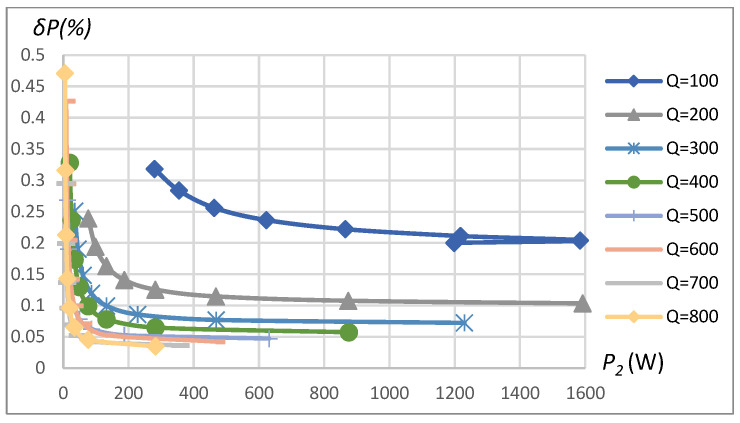
*δP* vs. transferred power *P_2_* and vs. quality factor *Q*. SS compensation topology, rectangular source, non-linear load, 16-bit ADC.

**Figure 16:**
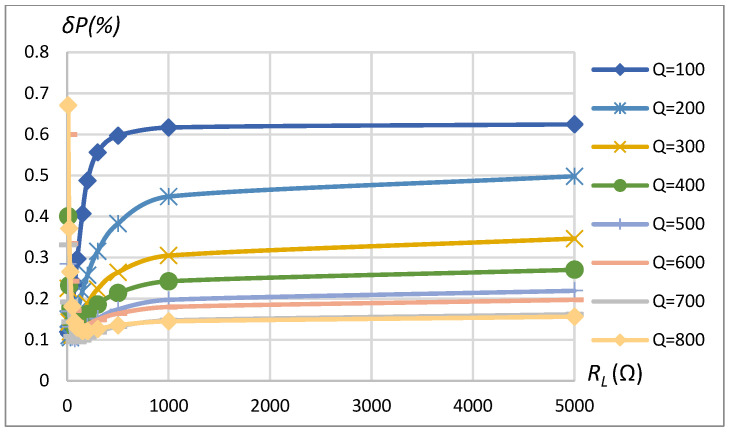
*δP* vs. load resistance *R_L_* and vs. quality factor *Q*. SS compensation topology, rectangular source, non-linear load, 16-bit ADC, k=0.25.

**Figure 17:**
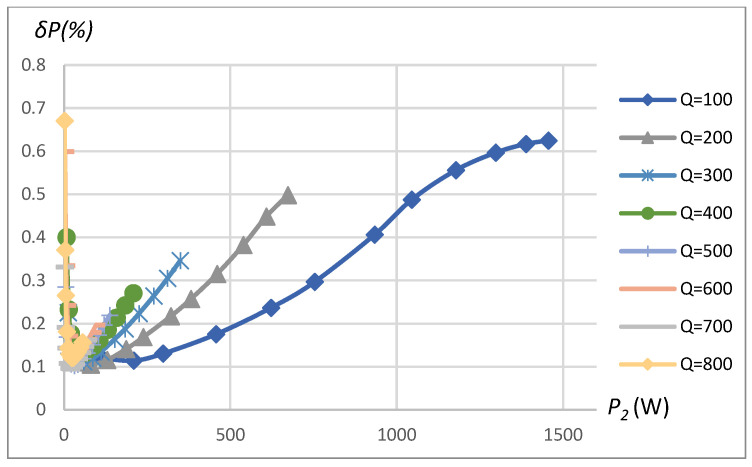
*δP* vs. transferred power *P_2_* and vs. quality factor *Q*. SS compensation topology, rectangular source, non-linear load, 16-bit ADC, k=0.25.

The influence of coupling coefficient, coil’s quality factor, and transferred power (or load resistance) on the assessment error of transferred power in the case of 16-bit quantization is shown in Figure 14, Figure 15, Figure 16 and Figure 17 for the SS compensation topology. The maximum error in the considered range of *k*, *Q*, and *P*_2_ for SP topology and also considering 12-bit quantization is summarized in Table 2. The obtained results indicated that all the influencing factors contribute to the assessment error *δP*. In a real-world environment, the particular value of these influencing factors cannot be known precisely because of the manufacturing tolerance of components and the mutual position of primary (stationary) and secondary (on EV) coils, as well as a load connected to the secondary coil. Nevertheless, it is possible to predict the value of the maximum possible assessment error if influencing factors stay within some of the most advanced known ranges.

The transferred power measurement error depends on the measured power, which, on the other hand, is a function of *k* and *R_L_*. Figure 15 is produced by changing *k* with constant *R_L_*, and Figure 17 is produced by changing *R_L_* with constant *k*. The transferred power corresponding to the combination of *k* and *R_L_* is shown on the horizontal axis in the plots of Figure 15 and Figure 17. Eventually, all the error dependencies on influencing factors (Figure 9, Figure 10, Figure 11, Figure 12, Figure 13, Figure 14, Figure 15, Figure 16 and Figure 17) were summarized in Table 2 by providing error limits with respect to typical ranges of influencing factors.

The results indicate that the method errors are in the acceptable range. Its operation in WTP systems with SS compensation topology is associated with a slightly lower range of error (*δP* less when 0.7%) in comparison to SP compensation topology (*δP* less when 1.1%). To assure low errors in a very wide range of transferred power, higher-bit ADC must be used (Table 2, 12-bit, and 16-bit cases). Research results indicate error ranges associated purely with the method itself and the quantization resolution (by an ideal ADC). The total error of the power measurement system will depend on many other factors, including the precision of voltage and current sensors, analog circuitry, operational conditions (temperature effects, noises), etc.

## 7. Discussion

The proposed measurement method targets enhancing the consumer experience in terms of fair billing for the energy acquired. The availability of techniques and their standardization, including verification methodologies, is vital for the wider adoption of wireless charging, especially in electrical transport systems. Consumers are very sensitive regarding the charging infrastructure of EVs. One of the key elements of charging infrastructure is the implementation of a certified metering system. The implementation, to a major extent, concerns EV and CS instrumentation manufacturers. In our proposal, the metering equipment that has to pass metrological verification is hosted in the CS rather than on the EV. This mapping of the measurement components reflects the current situation with petrol stations containing volume meters used for billing. Current and voltage sensing technologies are mature and well established and can be utilized in wireless CS. On the other hand, digital devices used to calculate active and reactive power are well developed for the fundamental frequency of the electrical distribution grid (50/60 Hz). WPT standard frequencies are in the range of 100 kHz. Therefore, the demand for digital signal processing hardware and software, probably involving designs of Application Specific Integrated Circuits, will arise following the validation of the proposed method.

While the metering equipment does not improve the energy efficiency itself, the data acquired and analyzed by both manufacturers and consumers will contribute to the optimization of onboard chargers’ efficiency, aiming to reduce charging costs. Supplementary consumed energy measurement at the receiver side (part of EV) and its comparison to the energy delivered from the transmitter coil (part of CS) may be used to reveal excessive energy losses, which may impact the safety of humans due to the exposure and absorption of electromagnetic radiation.

Another potential application of the suggested transferred energy measurement method could be related to EV alignment and positioning towards a single charging coil or a series of road-installed coil systems for dynamic wireless charging. Continuous data transmission about the delivered power to EVs can provide input to autonomous driving or driving assistance control to fine-tune EV positions similarly to image-based traffic line tracking. The primary method’s application is metering of power over MRC WPC system energy transferred to the receiving EV. Therefore, the metrological aspect was taken into account by designing the method in such a way that any metrologically certified meter is unnecessary onboard an EV. The technique could be applicable in other industries where the need to avoid a meter in the receiving equipment is not that critical. When a user energy meter is acceptable in the receiving part, it should be used because it can measure the energy received directly instead of using reflected impedance, as in our approach. Whereas in the case of EVs, the metering equipment is preferable in the technical infrastructure of the energy provider (similar to gasoline stations or contact EV charging stations). If the metering equipment is installed in an EV, then its owner would become responsible for periodic metrological calibration, probably at the time of the car’s technical inspection. Moreover, the metrological verification and/or calibration will significantly contribute to the cost of car maintenance. To add to that, depending on the country’s regulations, metrological verification might be requested after road accidents.

## 8. Conclusions

Wireless charging technology is beneficial and promising for EV charging applications. It is seen as a natural part of future sustainable roads for electric mobility in combination with energy harvesting methods and other modern technologies. Wireless charging stations, regardless of their implementation, whether conventional on-grid or off-grid, powered by energy generated from renewable sources, require proper transferred energy metering methods, ensuring that consumers are not billed for losses in the charging stations.

The proposed transferred power measurement method in wireless charging systems requires metrologically verified measurement of current and voltage only in the circuit of the transmitting (primary) coil. Applying the method, the customer of an EV can be billed only for the energy transferred to the receiver, excluding energy losses in the transmitter.

Modeling of the method, assuming a typical range of coil coupling coefficient, coil quality, and transferred power, is performed, aiming to determine the maximum possible transferred power assessment error. It was found that in the case of rectangular waveform driving of the primary coil and assuming a typical nonlinear load consisting of a diode rectifier, the active transferred power assessment error does not exceed 0.7% in the SS compensation topology and does not exceed 1.1% in the SP compensation topology if 16-bit resolution analog-to-digital conversion is used for current and voltage quantization. The total power measurement uncertainty can be estimated by combining the assessment error of the method with errors of current and voltage magnitude and phase measurement in the circuit of the primary coil. The limits of power assessment error were obtained in the range of coupling coefficient from 0.05 to 0.4, coil quality from 100 to 800, and power received at the secondary coil from 5 W up to 1.5 kW.

The future development will include hardware and digital signal software prototyping of the implementation of the method, followed by test procedures seeking to determine performance in the range of typical charging powers, types of loads, coil configurations, SS and SP compensation topologies, and vicinity of objects that may absorb the transmitted energy. Real-time transmitted power and energy measurement is the next step to be demonstrated in order to grade the computational load requirements for digital signal processing devices (microprocessors, programmable logic, application-specific integrated circuits, etc.).

## Figures and Tables

**Table 1:** Values and formulas for their calculations of parameters used in simulation.

Quantity/Parameter	Value/Expression
$U_{source}$	100 V
k	0.25 *
f	85 kHz
N	3
$f_{s}$	f·200 samples/s
$R_{p}$	1 Ω
$R_{s}$	1 Ω
$R_{DSon}$	0.1 Ω
$R_{L}$	75 Ω *
$R_{source}$	0.05 Ω
$L_{p}$	$\frac{R_{p}Q}{\omega }$
$L_{s}$	$\frac{R_{s}Q}{\omega }$
$C_{p}$ (SS topology)	$\frac{1}{\omega^{2}L_{p}}$
$C_{p}$ (SP topology)	$\frac{1}{\omega^{2}\left(L_{p}-M^{2}/L_{s}\right)}$
$C_{s}$	$\frac{1}{\omega^{2}L_{s}}$
$C_{3}$	100 μF

* Parameters vary in particular research cases.

**Table 2:** Transferred power maximum relative (in percent) and absolute (in Watt) assessment error $\delta P_{max}$ in the considered range of influencing factors.

Topology	Driving Waveform	Quantization, Bit	Load Type	*k* Range	*R_L_* Range, kΩ	Coil Quality *Q*	$\delta P_{max}$, %	$\delta P_{max}$, W
SS	sin	none	linear	0.05–0.4	75	100–800	6.3 × 10^−4^	5.8 × 10^−3^
SS	sin	none	linear	0.25	0.01–5	100–800	1.6 × 10^−3^	8.2 × 10^−3^
SP	sin	none	linear	0.05–0.4	75	100–800	0.1	0.7
SP	sin	none	linear	0.25	0.01–5	100–800	2.9 × 10^−2^	0.4
SS	rectangular	none	non-linear	0.05–0.4	75	100–800	0.4	2.1 × 10^−2^
SS	rectangular	none	non-linear	0.25	0.01–5	100–800	0.6	9.1
SP	rectangular	none	non-linear	0.05–0.4	75	100–800	1.0	6.2
SP	rectangular	none	non-linear	0.25	0.01–5	100–800	0.6	1.0
SS	rectangular	12-bit	non-linear P2 (5 W–1.6 kW)	0.05–0.4	75	100–800	2.3	0.1
SS	rectangular	12-bit	non-linear P2(2 W–1.53 kW)	0.25	0.01–5	100–800	4.4	8.3 × 10^−2^
SP	rectangular	12-bit	Non-linear P2 (0.54 kW–1.58 kW)	0.05–0.4	75	100–800	1.0	6.2
SP	rectangular	12-bit	non-linear P2 (44.9 W–1.59 kW)	0.25	0.01–5	100–800	0.6	3.4 × 10^−3^
SS	rectangular	16-bit	non-linear P2 (5 W–1.6 kW)	0.05–0.4	75	100–800	0.5	2.3 × 10^−2^
SS	rectangular	16-bit	non-linear P2 (2 W–1.53 kW)	0.25	0.01–5	100–800	0.7	1.3 × 10^−2^
SP	rectangular	16-bit	non-linear P2 (0.54 kW–1.58 kW)	0.05–0.4	75	100–800	1.0	10.7
SP	rectangular	16-bit	non-linear P2 (44.9 W–1.59 kW)	0.25	0.01–5	100–800	0.6	1.0

## Data Availability

Data are contained within the article.
